# Microextraction Technologies as Exposomic Sensors for On-Site Environmental Air Monitoring of Volatile Organic Compounds: A Review of Commercially Available Technologies

**DOI:** 10.3390/molecules31040580

**Published:** 2026-02-07

**Authors:** Stefano Dugheri, Nicola Mucci, Ilaria Rapi, Giovanni Cappelli, Ettore Guerriero, Fabio Cioni, Domenico Cipriano, Ivana Stanimirova, Veronica Traversini, Antonio Baldassarre, Riccardo Gori

**Affiliations:** 1 Department of Life Sciences, Health and Health Professions, Link Campus University, via del Casale di San Pio V 44, 00165 Rome, Italy; 2 Department of Experimental and Clinical Medicine, University of Florence, Largo Giovanni Alessandro Brambilla 3, 50134 Florence, Italyilaria.rapi@unifi.it (I.R.); veronica.traversini@unifi.it (V.T.);; 3 Division of Occupational Medicine, Careggi University Hospital, 50134 Florence, Italy; 4 Institute of Atmospheric Pollution Research (IIA), National Research Council (CNR), Strada Provinciale 35d n. 9, 00010 Montelibretti, Italy; ettore.guerriero@cnr.it; 5 Regional Agency for Environmental Protection of Tuscany, via del Ponte alle Mosse 211, 50144 Florence, Italy; 6 RSE SpA—Energy System Research, 20134 Milan, Italy; 7 Institute of Chemistry, University of Silesia in Katowice, 9 Szkolna Street, 40-006 Katowice, Poland; 8 Department of Civil and Environmental Engineering, University of Florence, via di Santa Marta 3, 50139 Florence, Italy

**Keywords:** Microextraction Technologies, exposomics, volatile organic compounds, air monitoring

## Abstract

Microextraction Technologies (METs) have emerged as pivotal exposomic sensors for the on-site monitoring of Volatile Organic Compounds (VOCs) in ambient air. By integrating sampling and sample preparation into a single step, METs provide solvent-free, miniaturized, and field-deployable solutions that align with the principles of green analytical chemistry. This review critically examines fourteen commercially available METs, selected for their demonstrated analytical performance, commercial accessibility, and validation in real-world environments. These devices represent the current state of practice in exposomics, enabling both short-term hotspot detection and long-term exposure assessment. Particular attention is given to their compatibility with transportable and portable detection platforms, including vehicle-mounted and hand-held gas chromatography/mass spectrometry systems, where METs function as front-end concentrators that enhance sensitivity and spatial resolution. This review further discusses emerging applications in wearable formats and unmanned aerial vehicles, underscoring the role of METs in bridging laboratory-grade precision with field-based exposome research. By situating METs within the broader exposomic workflow of sampling, detection, and interpretation, this work identifies current technological gaps and outlines priorities for advancing robust, scalable, and environmentally sustainable exposure assessment strategies.

## 1. Introduction

Environmental factors, particularly environmental pollutants and lifestyle behaviors, are among the main drivers of chronic non-communicable diseases, which now dominate global mortality. The chemical industry transforms raw materials into more than 350,000 distinct substances and products [1]. Consequently, unconsented exposure to these harmful substances constitutes a human rights violation. Du et al. [2] report an average lifetime cancer risk of 2.27 × 10^−4^, equivalent to an additional 2.27 cases per 10,000 exposed individuals, attributable to Hazardous Air Pollutants (HAPs). The Global Burden of Disease study reported that outdoor air pollution will cause approximately 6 to 9 million premature deaths annually by 2060. The associated global welfare losses, derived from individual willingness-to-pay estimates for reducing mortality risk, are expected to rise from USD 3 trillion in 2015 to USD 18–25 trillion by 2060. In addition, the welfare costs due to pain and suffering from pollution-related illnesses are likely to increase from around USD 300 billion in 2015 to about USD 2.2 trillion by 2060 [3]. The concept of the exposome was first proposed by Christopher Wild in 2005 [4], and the term encompasses the totality of human environmental exposures (defined as all non-genetic factors) from conception onwards. This framework complements the genome and emphasizes the urgent need for more comprehensive and precise environmental exposure data.

The general external exposome encompasses air quality determinants such as Volatile Organic Compounds (VOCs) [5]. VOCs are commonly defined as carbon-containing organic compounds with a boiling point below 373.15 K at 101 kPa and a vapor pressure above 13.3 Pa at 25 °C [6]. The EU Solvents Directive (1999/13/EC) provided a complementary criterion, defining VOCs as organic compounds with a vapor pressure of at least 10 Pa at 20 °C [7]. Emitted from diverse anthropogenic and natural sources, VOCs have attracted growing attention due to their widespread release and significant environmental and health impacts; they can also generate odor nuisance, adversely affecting quality of life [8]. Among VOCs, 187 have been classified as HAPs because of their demonstrated potential to pose serious risks to human health and ecosystems [9,10]. VOCs’ effective environmental quality assessment and pollution control depend fundamentally on analytical chemistry, which supplies the methods and techniques necessary to detect and quantify both inorganic and organic pollutants.

The concept of the exposome has seen a rapid growth in the literature, with PubMed results increasing sharplyfrom 202 between 2005 and 2015 to 3404 in 2015–2025. This expansion reflects two primary motivations: first, the simultaneous assessment of multiple environmental exposures promises a more comprehensive evaluation of their collective impact on human health; second, a high-precision characterization of environmental exposures enables integrated studies of gene–environment interactions. Several large-scale exposome projects have emerged, particularly in Europe, such as the HELIX initiative, which integrates early-life environmental hazards, ranging from chemical to physical stressors, and correlates them with child health, growth, and respiratory outcomes [11]. Another important project is EXPOsOMICS, which develops novel exposure assessment methodologies for air and water pollutants, linking them to the molecular biomarker effect [12]. The Heals consortium aims to construct individualized internal exposomes by combining omics data with biochemical biomonitoring [13]. In the United States, the HERCULES program provides infrastructure and expertise to advance exposome tool development, validation, and application in epidemiological research [14].

Since the 1960s, the introduction of comprehensive environmental regulations has driven an escalating demand for the quantitative analysis of environmental samples [15]. Such data underpin monitoring of air, water, and soil quality; the evaluation of contaminants distribution over space and time; the assessment of contamination severity; and tracking of remediation effectiveness. Currently, the exposome paradigm offers an integrative framework to consolidate fragmented environmental exposure data and to account for multifactorial stressors in disease etiology [16,17]. To operationalize the exposome in population studies, investigators have adopted geographic information systems, remote sensing platforms, smartphone-based personal exposure sensors, and high-throughput miniaturized air samplers [18]. On-site, continuous analysis of chemical stressors, conducted in situ rather than in centralized laboratories, shows its potential in exposomics, thanks to the accurate and timely assessment of environmental hazards [19]. Moreover, portable green analytical tools eliminate errors, delays, and costs associated with sample transport and storage, and lead to a reduction in the use of solvents by combining the sampling phase with the sample preparation phase [20]. Recent technological innovations have encompassed field-deployable separation and detection systems, such as compact Gas Chromatographs (GCs), Liquid Chromatographs (LCs), and Mass Spectrometers (MSs), alongside continuous monitoring devices and point-of-care platforms. Over the past decade, the extensive miniaturization of these instruments has driven significant reductions in operational time and cost [21,22,23,24,25,26,27,28]. Portable MS units are gaining traction and are set to transition from research settings to control laboratories, enabling on-site and at-line analyses. Today, both vehicle-portable and man-portable GC, LC, and MS systems are advancing rapidly: vehicle-portable platforms are evolving toward temporally and spatially resolved mapping once limited to stand-off methods, while man-portable formats are shrinking from bulky “luggable” to wearable backpacks and even handheld units. Portable, miniaturized sampling tools, such as Microextraction Technologies (METs) and GC, LC, and MS, offer the real-time, high-resolution chemical measurements essential for comprehensive multi-stressor evaluation and integrated gene–environment analyses in large-scale population studies, as required by exposomics.

The literature selection presented in this analysis was conducted by consulting the PubMed, Scopus, and Web of Science databases, examining contributions published from 2000 to 2025. The selection criteria favored studies reporting validations in real-world scenarios, the commercial accessibility of devices, and on-site analytical performance. In order to ensure a comprehensive overview of the principal technologies currently available on the market, the research was extended to gray literature, including application notes and technical data sheets provided directly by manufacturers (e.g., Markers International, Gerstel, Restek, and Supelco).This review situates METs as exposomic sensors applicable across multiple matrices, including air, water, and soil, where Volatile and Semi-Volatile Organic Compounds represent critical exposure pathways. By critically examining fourteen commercially available devices, selected for their analytical performance and field validation, we clarify how METs interact with portable and transportable detection systems to enable both short-term hotspot identification and long-term exposure assessment. In doing so, this review highlights their position within the exposomic workflow—sampling, detection, and interpretation—and identifies current technological gaps together with priorities for advancing robust, scalable, and environmentally sustainable exposure assessment.

## 2. Results and Discussion

Urban air quality continues to be a pivotal determinant of public health, with even short-term exposures to fine Particulate Matter (PM_2.5_) and particle-phase VOCs showing measurable impacts [29,30]. Primary pollutant concentrations can vary substantially over short spatial scales with a variability which is often poorly represented by routine monitoring networks but is critical for any accurate exposure assessment, air quality management, and environmental equity [31,32]. Technological advancements over recent decades have facilitated continuous monitoring and the implementation of Field Analytical Methods (FAMs), combining portable instrumentation, mobile laboratories, and regulatory monitoring networks such as the EPA Air Quality System (AQS) [33]. Frameworks such as the Triad Approach, integrating systematic planning, dynamic workplans, and field-based measurement technology, seek to optimize site assessment while reducing uncertainty in environmental decision-making [34]. Despite these advances, regulatory networks remain limited in their capacity to capture fine-scale spatiotemporal variability, particularly for primary pollutants, with implications for epidemiological studies and population exposure estimation [35,36,37,38,39]. To address these gaps, complementary approaches have been developed, including satellite remote sensing, chemical transport models, land use regression, and direct personal exposure measurements [40]. Each method presents distinct advantages and limitations, emphasizing the importance of integrating multiple approaches to resolve local pollution gradients and micro-scale variations [41]. Mobile monitoring using professionally driven fleet vehicles or public transport, alongside dense networks of low-cost sensors, has proven to be effective in mapping neighborhood-level differences and persistent hotspots within urban areas [42,43,44,45]. These strategies enhance the spatial resolution of air quality data, improve the characterization of human exposure, and provide a robust foundation for interpreting long-term environmental and health outcomes.

### 2.1. Classification METs

**Figure 1:**
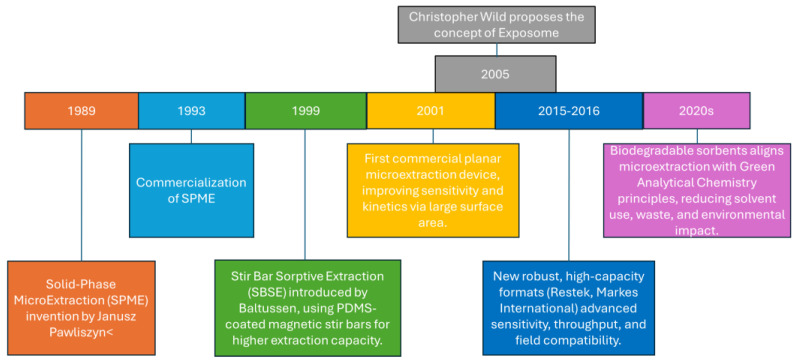
Timeline of METs development with respect to the concept of the exposome.

Across environmental matrices, including air, water, and soil, sampling and sample preparation typically account for 70–80% of the total analytical time. This finding has driven the development of solutions aiming to minimizing both the time and labor required during the preparation phase, while simultaneously addressing the need to enhance the sensitivity of various analytical methods [46]. By merging sampling and preparation into a single operation, METs streamline exposomic workflow sampling, detection, and interpretation, while reducing solvent use and enabling near real-time measurements. Although METs have historically been associated with grab sampling (instantaneous sampling), technological advances now allow for proper exposomic assessments through TWA (Time-Weighted Average) monitoring. Devices with planar geometries such as TF_SPME or silicone wristbands act as passive samplers, allowing exposure to be integrated over hours, days or weeks. This approach allows daily variations to be leveled out and the impact of transient interferents to be reduced, providing a measure of chronic contamination that is essential for epidemiological studies. The typical procedure for monitoring complex samples encompasses multiple stages: sampling, sample preparation, separation, quantification, statistical evaluation, and decision-making analysis. Analytical efficiency and accuracy depend critically on selecting a sampling technique tailored to the matrix and target compounds, since this choice determines sensitivity, selectivity, and regulatory compliance. Thus, defining an appropriate monitoring approach requires a comprehensive understanding of the available sampling and analytical methods. Samples collected for compliance purposes are often repurposed beyond their original intent, enabling the identification of pollution sources, the assessment of control measures’ effectiveness, and the evaluation of the relationships between pollution levels and public health outcomes. Since their introduction in 1989 (Figure 1), METs have evolved to meet these requirements, offering robust formats applicable across diverse environmental matrices and reducing both analysis time and uncertainty.

METs, by integrating the sampling and sample preparation steps into a single step, support the entire exposomic analytical workflow: sampling, detection, and interpretation. Furthermore, miniaturization and the reduced use of reagents (consistent with the principles of green analytical chemistry) are fundamental aspects for achieving near real-time measurements. The analytical performance is influenced by factors such as the volume and type of sorbent phase, device geometry, and extraction efficiency for target analytes, affecting both sensitivity and capacity.

Recent classifications of METs as exhaustive or non-exhaustive have practical implications for calibration strategies, sampling duration, and suitability for field deployment [47]. Conversely, earlier studies, such as those by Nerin et al. [48], classified METs according to conventional Solid-Phase Microextraction (SPME) fiber and in-tube developments, while Mehdinia et al. [49] distinguished between static and dynamic techniques. Nonetheless, the classification of METs methods remains neither fully clear nor comprehensive. This study proposes a coherent terminology for describing METs. To this end, terms recommended by the International Union of Pure and Applied Chemistry (IUPAC) for analytical Solid-Phase Microextraction (SPME) were adopted [50]. A classification system that categorizes METs into four groups based on geometry was developed, namely planar, in-tube, coated bar–disk, and SPME (Table 1). Similarly, METs’ characteristics are being evaluated, including the use of porous or liquid phases, active or passive samplers, short- and/or long-term sampling, personal and/or area sampling, and exhaustive or non-exhaustive approaches. In addition, considering the exposomic framework, a brief evaluation was carried out on their compatibility with drones, wearable devices, and automated collection boxes, which represent the most widespread and ready-to-use solutions for greater spatial and temporal coverage of contaminant dispersion. Drones enable targeted spatial and vertical sampling of airborne contaminants across heterogeneous landscapes and hard-to-reach locations, while wearables are optimized for personalized exposome assessment, capturing individual time-resolved exposures that reflect microenvironments and activities, and the latter, stationary, programmable samplers, provide continuous or scheduled sampling at fixed points, supporting long-term temporal trends and networked spatial coverage [51,52]. For environmental monitoring, geometry is critical, since sampler placement and positioning influence uptake kinetics and representativeness across air, water, and soil matrices. As noted, METs can be developed in various formats, but thin-film, high-surface area geometries are proposed as the optimal choice for environmental analysis in both laboratory and field settings.

This list above provides the framework for evaluating the commercially available METs discussed in the following sections, highlighting how geometry, sorbent chemistry, and sampling mode could determine their role as exposomic sensors (Table 1).

#### 2.1.1. Planar METs

The Thin-Film MET (TF-MET) has been commercially introduced by GERSTEL (Mülheim an der Ruhr, Germany) as TF-SPME as a planar microextraction device. Introduced in 2001 [53], it has since been employed for various gas and liquid chromatography applications [54]. Its large extractive-phase volume and thin-film geometry maximize the surface area, enhancing the analyte uptake and accelerating extraction kinetics [55]. The enhanced sensitivity of the TF-MET technique can be understood by applying the fundamental mass balance rule to SPME at equilibrium conditions [56]. Equation (1), derived from the mass balance under equilibrium conditions, defines the intrinsic relationship between the amount of analyte (the number of moles) extracted into the extractive phase at equilibrium (n_e_^eq^) and its original concentration ($C_{s}^{0}$) in the sample. In this equation, Kes denotes the distribution constant of the analyte for the extractive phase and sample matrix, with Vs and Ve referring to the volume of the sample and extractive phase, respectively. Ultimately, this equation indicates mathematically that an increase in the volume of the extractive phase results in improved analytical sensitivity.

(1) $$n_{e}^{eq}=\frac{K_{es}V_{s}V_{e}}{K_{es}V_{s}+V_{s}}C_{s}^{0}$$

In the abovementioned scenario, the equilibrium extraction time (t_e_), defined as the time required to extract 95% of the equilibrium amount, can be calculated by Equation (2); where Ds, b, and δ refer to the diffusion coefficient of analyte in the sample, the thickness of the extractive phase, and the thickness of the boundary layer, respectively. This equation illustrates that for a given amount and type of extractive phase, a faster extraction equilibrium can be achieved when a thin layer of extractive phase is used.

(2) $$t_{e}=3\delta K_{es}(b)/D_{s}$$

Moreover, Equation (3) implies that the initial extraction rate, denoted as extracted amount (dn) per given time (dt), is directly proportional to the surface area of the extractive phase (A). Therefore, if a relatively large volume of extractive phase is spread over an ultra-thin layer, there will be a larger interface between the sample and the extractive phase, resulting in faster extraction kinetics.

(3) $$\frac{dn}{dt}=\left(\frac{D_{s}A}{\delta }\right)C_{0}$$

Advances in TF-MET include specialized holders and samplers designed for diverse environments, such as rigid membranes for stability, Time-Weighted Average (TWA) devices for long-term monitoring, and robust blades for extreme conditions [57,58]. Hydrophilic–Lipophilic Balance (HLB) particles have been adopted to improve affinity for polar analytes [59]. TF-SPME devices are compatible with both gas and liquid chromatography, highlighting the critical role of sampler design in optimizing sensitivity and reproducibility [58]. Quintanilla et al. [60] applied a cellulose triacetate film doped with multiwalled carbon nanotubes for the monitoring of chlorpyrifos, tonalide, and triclosan in river water, and afforded limits of detection between 0.2 and 0.5 µg L^−1^, recoveries of 90–104%, and intra-film precision (RSD) < 7%. Notably, no significant matrix effects were observed for chlorpyrifos and tonalide, underscoring TF-MET’s robustness in complex environmental matrices. In the context of drinking water safety, thin-film SPME membranes coated with Hydrophilic–Lipophilic Balanced/polydimethylsiloxane phases have been paired with GC×GC–TOF MS to screen halogenated Disinfection By-Products (DBPs) at sub-ppb levels. The planar sorbent provided a 2.5-fold increase in extraction efficiency over fiber SPME [61]. Paper-based TF-MET patches offer an even more user-friendly format. Poojary et al. [62] proposed the use of disposable patches with divinylbenzene/PDMS/CNT coatings on cellulose paper for the quantification of 4-chlorophenol in water. Direct exposure for 10 min yielded a calibration range of 100–10,000 ng mL^−1^ and a limit of detection of 10 ng mL^−1^, validating the rapid field screening of this priority pollutant at regulatory threshold concentrations. Planar METs have also enabled faster and more sensitive air monitoring of explosives and other analytes, particularly when coupled with ion mobility spectrometry or advanced ionization techniques, including DART, DESI, SELDI, and MALDI [63,64,65,66].

Coated Blade Spray (CBS), developed by Gómez-Ríos and Pawliszyn [67], allowed for a direct and real-time analysis with minimal sample preparation, achieving low-picogram detection limits within three minutes and compatibility with portable and benchtop Mass Spectrometers [68]. Beyond its application to relatively simple matrices, CBS has been successfully tested on complex environmental and biological samples, where the selective polymer coating enables the efficient enrichment of target analytes. This capability demonstrates its potential as an exposomic sensor across diverse matrices, allowing direct coupling to mass spectrometry. Hu et al. [69], using CBS, developed a high-throughput and automated sample preparation method with the use of a 96-well plate system, enabling analysis in water of 21 drugs of abuse within 1 min per sample, while using only 8 µL of organic solvent for desorption and atmospheric pressure MS detection; coupling CBS and MS led to achieve low limits of detection from 1.5 to 9.0 ng L^−1^.

According to Yu et al. [70], CBS, functionalized with biocompatible polymers or hybrid nanosorbents, achieves comparable or lower limits of detection (0.01–0.5 µg L^−1^) for phenolic and polyaromatic pollutants than TF-MET, while offering superior mechanical stability and rapid thermal desorption directly within GC inlets. The planar, rigid format simplifies handling, facilitates automation, and withstands field conditions (temperature swings, biofouling, mechanical shock).

Taken together, TF-MET and CBS epitomize the ongoing miniaturization and greening of environmental analysis. Their high extraction capacity, minimal solvent use, and compatibility with advanced chromatographic–mass spectrometric techniques position them as front-line tools for on-site monitoring of legacy and emerging contaminants.

#### 2.1.2. In-Tube METs

In parallel with planar TF-MET, in-tube or in-needle METs have been developed, offering exhaustive or non-exhaustive sampling, depending on the device design. Their compact geometry and compatibility with automated workflows make them particularly relevant for exposomic monitoring [71]. The first non-exhaustive in-tube device, the Solid Phase Dynamic Extraction SPDE^TM^ Magic Needle (Chromtech, 2000, Bad Camberg, Germany), consists of stainless-steel needles coated with sorbent and activated carbon, providing a larger stationary-phase volume than conventional SPME fibers and enabling automated, high-efficiency extraction using gas-tight syringes [72,73]. SPDE operates as a dynamic, non-equilibrium technique in which analyte enrichment is achieved through repeated suction cycles. However, an initial incubation period (typically 10–30 min at 60–70 °C) is required to promote analyte release into the headspace before dynamic extraction begins [74,75]. This dynamic approach confers a high enrichment capacity, with sensitivities reported up to ten-fold greater than static headspace methods for fire accelerant determination. Moreover, SPDE exhibits an exceptionally wide linear dynamic range, spanning nearly five orders of magnitude with PDMS coatings and exceeding six orders for VOCs in groundwater, underscoring its suitability for complex exposomic matrices.

Needle Trap Microextraction (NTME), introduced in 2001 by Pawliszyn’s group, serves as an exhaustive particle sampler, using commercial sorbents such as PDMS/DVB, Carboxen, Carbopack X, Tenax TA, and polymer beads. Combining various multiple sorbents or using nanoporous silica further enhances its performance [76,77,78]. Recent developments in Needle Trap Devices (NTDs) focus on advanced sorbent materials, including hybrid polymers and Metal–Organic Framework (MOF) coatings. For example, MIL-100(Fe) has been investigated as a MOF sorbent, offering enhanced selectivity and stability under field conditions. In addition, the robustness of NTDs has been confirmed: Anticó demonstrated that 22-gauge NTDs equipped with side-hole configurations and a thermally desorbed in-injector at 300 °C yielded reproducible sampling (RSD < 8%) and LODs of 0.01–0.1 µg m^−3^ for VOCs in indoor and outdoor air, with no carryover after 48 h of storage at 4 °C [79]. Direct thermal desorption within the injector preserves compound integrity and enables reproducible on-site airborne monitoring, eliminating the need for auxiliary gas lines or valves. This robustness is critical for exposomic studies that require reliable sampling under variable environmental conditions. In remote atmospheric campaigns, portable GC-MS platforms integrated with NTME sampling have quantified monoterpenes, aldehydes, amines, and anthropogenic VOCs at forest sites: Barreira et al. [80] reported that needle trap sampling followed by field-deployable GC-MS detected monoterpenes at 0.1–1 ppb, correlating diurnal emission patterns with particle number concentrations and illustrating the system’s utility for real-time ecosystem monitoring. Passive sampling variants of needle traps also exist [79]. Similarly, the NeedlEX device (Shinwa Chemical Industries, Kyoto, Japan) allows for repeated, automated air sampling of alcohols, organic solvents, amines, and fatty acids directly via GC injection without additional equipment [81]. In-Tube Extraction (ITEX) is a dynamic sampling method, often considered a cost-effective alternative to the purge and trap technique for enriching VOCs from aqueous samples. As a high-capacity enrichment technique (similar to SBSE), it provides high sensitivity; for example, the LOD for VOCs in honey has been reported in the range of 0.8 to 47 ng/g. ITEX demonstrates excellent precision and repeatability, with a relative standard deviation typically around 3% [82].

ITEX-InTube Extraction (CTC Analytics, Zwingen, Switzerland, 2008) uses a syringe-based microtrap packed with adsorbents (Tenax, Carbopack, Carbosieve, molecular sieve) to concentrate target compounds from air, which are then thermally desorbed directly into the GC injector. The ITEX-DHS approach employs a gas-tight syringe fitted with a micro-trap of adsorbent packing to repeatedly withdraw headspace from aqueous samples, enabling the dynamic enrichment of odorants such as geosmin, 2-methylisoborneol, 2,4,6-trichloroanisole, and isoborneol at sub-ppt concentrations. Under optimized conditions (60–100 repeated strokes, trap desorption at 250 °C), LODs down to 1–5 ppt were achieved, with linear ranges spanning three orders of magnitude and recoveries of 92–105% in river and drinking waters [83]. Full automation is achieved using modified software and autosamplers for preconditioning, internal standard addition, exhaustive extraction, and desorption [84,85,86].

Exhaustive METs include Sorbent Pens™ (Entech Instruments, Simi Valley, CA, USA, 2016), available in diffusive or active modes with a variety of sorbents for short- or long-term air sampling. PowerSorb^®^ (Action Europe, 2017) is a polymer-based cylinder designed for thermal desorption or solvent extraction, applicable as a passive sampler or SPME alternative, with applications ranging from human scent detection to VOC monitoring for health and environmental studies [87,88,89,90,91,92]. Supplied in pre-packed vials under inert gas, the 20 mm × 2 mm polymer rod can be directly immersed in aqueous samples or deployed for dynamic and passive headspace sampling of VOCs without any prior conditioning steps.

The Getxent^®^ microtube (Getxent, Biodesiv LLC, Switzerland) is a cost-effective polymer stick (3.8 × 30 mm) suitable for exposome analysis, compatible with thermal desorption below 100 °C [93,94]. Its simplicity and affordability make it attractive for large scale exposome studies, particularly where high sample throughput and minimal infrastructure are required. Taken together, in-tube METs provide versatile formats ranging from dynamic non-equilibrium enrichment (SPDE, ITEX) to exhaustive sorbent traps (NTME, Sorbent Pens). Their adaptability to complex matrices, compatibility with automation, and robustness under field conditions position them as essential tools for exposomic monitoring.

#### 2.1.3. Coated Bar–Disk METs

The oldest METs in terms of coated bar conception, the Stir Bar Sorptive Extraction (SBSE) technique, patented in the US in 2002 [95], was proposed by Baltussen in 1999 [96]. The device is a magnetic stir bar enclosed in a glass shell covered with a sorbent layer with a PDMS sorbent layer (0.5–1 mm), commercially available as Twister (Gerstel GmbH) [97]. These bars come in two sizes for different sample volumes, and in 2012, an Ethylene Glycol (EG) silicone sorbent phase was introduced to extend the range of extractable compounds [98,99,100]. SBSE is characterized by a very high extraction capacity thanks to the thick layer of PDMS used, with almost quantitative recoveries for nonpolar compounds (log Kow > 5). PDMS coatings exhibit high durability, remaining stable for at least 100 extraction cycles, allowing a large number of samplings with costs (around 100$ each SBSE). Concerning airborne pollutants, for Polycyclic Aromatic Hydrocarbons (PAHs), extending SBSE sampling durations to 60 min and coupling to thermal desorption GC–MS achieved sub-ng m^−3^ sensitivity. SBSEs are particularly well suited to the study of long-term environmental exposure and the monitoring of chronic contamination. Thanks to their high capacity and robustness, passive sampling provided by SBSEs offers an integrated measure of exposure that is not altered or limited by specific conditions on a given day, leveling out variables and reducing the weight of interferents in analytical evaluation. The MonoTrapTM—Monolithic Material Sorptive Extraction (MMSE) uses a porous silica monolithic hybrid surface functionalized with activated carbon, graphite, PDMS, and C18 groups, providing a high adsorption capacity. Extraction occurs primarily by adsorption with minimal solvent consumption and no need for preconditioning. Two adsorption chemistries are available: silica-like for nonpolar analytes and silica–carbon for enhanced polar compound retention. The innovative RGC18-TD adsorbent (graphitic carbon/ODS) offers high sensitivity; moreover, the implementation of vacuum-assisted extraction (Vac-HS-MMSE) reduces kinetic equilibrium times. It enhances sensitivity, with signal intensities increasing by roughly one order of magnitude for higher boiling analytes. This makes the technique effective for the analysis of odorous VOCs (aldehydes, ketones, alcohols) with very low odor thresholds (OTs), such as the compounds emitted in the environment by hot-mix asphalt during road surfacing [101].

MMSE devices are offered in rod and disk formats and can be reused after flushing [102,103,104]. In dynamic flow trials, 45 min sampling at 150 mL min^−1^ allowed for the determination of phenolic and nitro-aromatic compounds with LODs of 0.2–1.0 µg m^−3^, recoveries ranging from 65 to 92%, and RSDs under 12%. The rigid bar format facilitates direct thermal desorption in GC inlets (250–300 °C) and resists mechanical stresses encountered during portable or remote deployments [105]. The Magic Chemisorber (Frontiers Laboratories, 2004) is a solid-phase diffusive extraction device originally coated with a 500 µm PDMS layer, providing a high extraction capacity. Sampling is performed by direct immersion in aqueous samples or in the headspace phase. The device is effective for non-polar analytes such as PAHs and Polychlorinated Biphenyls (PCBs). Studies have reported excellent recoveries for PAHs and PCBs in water (96% to 110%) and a very low LOD of less than 2.7 ng L^−1^ for PAHs. Desorption occurs thermally in a pyrolysis–GC-MS system, with excellent reproducibility. In 2019, a PEG phase was introduced on the outer wall of a deactivated stainless-steel tube, enabling selective sampling of polar compounds [106,107]. Together, SBSE, MMSE, and the Magic Chemisorber exemplify the coated-bar MET family, which, thanks to their high sorbent capacity, mechanical robustness, and compatibility with thermal desorption, make them particularly suitable for long-term exposomic monitoring across different matrices, especially when integrated measures of chronic exposure are required.

#### 2.1.4. SPME METs

The SPME, first commercialized by Supelco in 1993 and improved in 2001 with a customized holder, has significantly influenced sample preparation in analytical chemistry by offering miniaturized, solvent-free workflows, short extraction times, low cost, and easy coupling to GC and LC [47]. These advantages have led to widespread applications in environmental studies and on-site analysis [108,109], particularly for VOC sampling, allowing for short- or long-term exposure, on-fiber derivatization, on-line sampling with autosamplers, and compatibility with field GC or GC-MS systems [110,111,112,113,114,115,116]. Advancements include Restek’s SPME Arrows (2015), which feature a larger-diameter, robust probe with increased sorbent volume for higher sensitivity and throughput, and Markes International’s HiSorb probes (2016), consisting of a thin inert rod wrapped with PDMS sorbent for non-exhaustive gaseous analyte extraction [117,118,119,120,121]. A direct comparison of the technologies reveals that extraction capacity is closely related to the volume of the extraction phase. While a conventional SPME fiber offers a limited volume (approximately 0.9 µL), Arrow SPME (3.8–11.8 µL) and SBSE/Twister (63–126 µL) provide a significant increase in analytical sensitivity, which is essential for detecting traces in complex matrices. In terms of operational robustness, Twister devices maintain stability for over 100 extraction cycles, while NeedlEx fibers are reusable for approximately 25–30 analyses. The use of Nitinol (NiTi) cores or StableFlex fibers has further improved mechanical durability compared with traditional fused silica. For environmental sampling, especially in polluted air, the use of porous adsorbent phases (e.g., Carboxen) can be subject to the displacement effect (competitive adsorption), in which high-concentration interferents compete for active sites, displacing target analytes. Recent advances in coating chemistry, encompassing inorganic materials (metal–organic frameworks, carbon nanotubes, graphene), organic polymers (polymeric ionic liquids, molecularly imprinted polymers), and hybrid nanocomposites, have substantially enhanced extraction capacity, thermal and solvent stability, and analyte specificity [122]. Although early SPME applications mostly involved laboratory analysis following field sampling, some recent studies have demonstrated its effectiveness for immediate on-site analysis. Gorecki and Pawliszyn showed SPME as a viable method for high-speed field GC separations [123]. Koziel et al. applied SPME for formaldehyde detection and other organic analytes in indoor air and field settings [124,125,126]. Recently, Strano et al. integrated conventional SPME fibers alongside photoionization (PID) and Metal-OXide (MOX) sensors on a small unmanned aerial vehicle to tackle airborne threats and fugitive industrial emissions. Using benchtop validation, multiple SPME coatings (e.g., PDMS, polyacrylate) were exposed to calibration atmospheres of VOCs and nerve agent simulants, yielding limits of detection in the sub-µg m^−3^ range (e.g., ≈0.1 µg m^−3^ for toluene and 0.05 µg m^−3^ for dimethyl methylphosphonate). The combined real-time response of the PID/MOX array guided flight paths to hotspots, while post-flight GC-MS analysis of the fibers provided compound-specific identification and quantitation, with recoveries between 85 and 110% and RSDs below 10% [127].

Other field GC-MS applications include the rapid detection of thermal degradation products from riot control agents [128]. A critical example of MET application in the exposome field is monitoring in the coffee processing industry, an environment characterized by the coexistence of carbon monoxide (CO), alpha-diketones, and bio-reactive dusts. In these scenarios, the grinding process represents the epicenter of risk, with diacetyl peaks that can exceed 9000 ppb. While photoionization detectors (PIDs) provide real-time data, METs coupled with GC-MS are indispensable for chemical speciation, allowing differentiation between diacetyl and 2,3-pentanedione, which is essential for the prevention of bronchiolitis obliterans. The adoption of analytically designed localized ventilation (LEV) has been shown to reduce exposure to al-alpha-diketones by up to 16 times.

### 2.2. Air Sampling and Monitoring

#### 2.2.1. Stationary Sampling

Recent advances in on-line SPME monitoring and automated sampling systems have significantly improved analytical workflows for environmental and field studies. While conventional stationary samplers provide accurate measurements at fixed locations, mobile laboratories positioned close to sampling sites enable on-line sampling, often via Teflon lines. Commercially available, fully automated miniaturized techniques rely primarily on three-axis (xyz) autosamplers, which have proliferated over the last decade, particularly when coupled with GC. These systems, featuring modularity and flexibility, support microextraction techniques for air sampling and can be equipped with accessories such as barcode readers, robotic syringe changers, decappers, heating and stirring modules, and LAN connectivity to enhance usability and productivity [129,130]. CTC Analytics AG pioneered commercial xyz autosamplers, releasing the first GC liquid autosampler (A200S) in 1986, followed by the PAL system in 1998 and subsequent expansions including the HTX PAL, PAL RTC, RSI, and LSI systems (2003–2014). Today, various companies, Leap Technology, Chromtech, Markes International, GERSTEL, KONIK Group, EST Analytical, Moduvision Technologies, and PAS Technology, produce customized autosamplers to meet diverse analytical needs and improve workflow efficiency. In parallel, portable sequential sampling devices have been developed for environmental pollution assessment, capable of remotely collecting gases, vapors, and Particulate Matter (PM) via tubes, filters, denuders, canisters, or bags, often in compliance with EPA speciation requirements. These systems are increasingly applied in industrial hygiene for area sampling. Examples include CEH DELT^®^, GasCheck™ (AMS Analitica, Pesaro, Italy), SASS™ (Met One Instruments, Grants Pass, OR, USA), URG-2000, Partisol^®^ Model 2300, GAS08/16, Echo TUBE, and MTS-32™ (Markes International, Bridgend, Wales (UK)). Innovative solutions such as ODORPREP^®^ (Lab Service Analytica, Anzola dell'Emilia, Italy) offer real-time, automated, remote-activated sampling of industrial odor emissions, compliant with European Standard EN 13725 [131], and supported by Horizon 2020 funding (Project OdorPrep, G.A. 756865). Complementing this, OdorBOT enables citizen reporting of odor nuisances via a Telegram™ interface, collecting and mapping real-time data for subsequent analysis [132].

#### 2.2.2. Mobile Sampling

Mobile and wearable sampling approaches have advanced individual exposure and exposome monitoring by enabling the high-resolution characterization of chemical interactions in diverse microenvironments. Mobile systems, including automobiles, drones, helicopters, or boats, allow the rapid deployment of SPME samplers or mobile GC systems with adapted power and carrier gas supplies. These roving systems provide high-speed, repeated measurements that capture transient events and map sharp spatial concentration gradients, including point sources [133,134,135]. On the road, vehicle-mounted SPME–GC systems have likewise been employed for high-resolution mapping of urban VOC plumes. One example includes Kore PTR 3c, equipped with Live-Drive Technology and Instavac Systems for near-instant VOC measurements, and drone-mounted TF-SPME devices for the on-site screening of pollutants in hard-to-reach water bodies [59]. METs represent a powerful tool for environmental and exposome assessment, as they are easily integrable with unmanned aerial vehicles (UAVs) or drones; miniaturization and generally no need for reagent consumption are crucial, as they help to drastically reduce overall mass and energy requirements.

Field studies and reviews have demonstrated the effectiveness of drone-compatible METs. Notable examples include a TF-SPME drone water sampler with a payload of about 12 g, coupled with a portable GC-MS for on-site screening, and an aerial SPME Arrow campaign that collected 135 samples at heights between 50 and 400 m and identified 48 VOCs [136].

Nowadays, the main challenges concern the need to further improve energy efficiency, reduce mass, and ensure the stability of portable analytical configurations (such as miniaturized GC-MS), that accompany METs analyses. Wearable samplers are deployed on participants or in microenvironments to assess inhalation exposure, using either active devices (pump-assisted quantitative sampling) or passive devices (time-integrated sampling via diffusion). Early developments include wearable SPME necklaces and pins for ketamine monitoring using ionic liquid coatings [137]. More recent innovations, such as the Fresh Air Wristband Clip, integrate PDMS sorbent bars and triethanolamine-coated pads to monitor VOCs, PAHs, and NO_2_ [138,139,140,141]. Sorbent membranes have also been incorporated into badges, brooches, and lapel diffusion tubes, enabling personal exposure assessment. Silicone wristbands have proven effective for the multi-hour to multi-week monitoring of environmental and occupational pollutants, including polybrominated diphenyl ethers, organophosphate flame retardants, and pesticide residues, and have been successfully applied across diverse populations and geographic locations. By integrating exposures over extended periods, these devices provide a more comprehensive picture of chronic contamination that is not distorted by day-to-day variability. In this way, transient fluctuations and analytical interferents are leveled out, reducing their impact on data interpretation and supporting long-term exposomic studies of environmental exposure. In one field deployment, participants wore silicone wristbands continuously for ten days; the analysis revealed a median of 43 unique chemicals per device, with individual analyte masses spanning 0.1–500 ng per band [52]. Recently, miniaturized electrochemical and optical sensors have been integrated into wearable badges, vests, or backpacks to provide real-time measurements of gases and particulates. Urban cohort studies using wearable PM2.5 monitors recorded personal exposures averaging 28 ± 12 µg m^−3^, approximately 23% higher than concurrent fixed-site measurements, highlighting substantial exposure variability during commuting and indoor–outdoor transitions [142].

#### 2.2.3. Transportable and Portable Instruments

Two complementary deployment strategies have emerged to bridge the gap between field sampling and laboratory analysis. Vehicle-based mobile laboratories, which require the installation of compact benchtop GC or LC systems in trailers or vans, maintain stable power supplies and controlled environmental conditions that closely replicate those of fixed laboratories. This approach dramatically reduces the time between sample collection and data generation, allowing complex GC-MS and LC-MS workflows to be carried out directly at the point of interest. At the same time, advances in microfabrication and power management have led to the development of truly portable chromatographs, which integrate pumps, capillary columns, detectors, and electronics within a single enclosure. According to Sharma et al. [143], such systems must operate autonomously for at least eight hours, which is ideal for monitoring occupational exposure, which normally refers to intervals or limits over eight hours, using battery or solar power; achieve rapid thermal equilibration; and resist extremes of temperature and humidity, dust, and mechanical shock. They must also deliver separations and sensitivities comparable to those of benchtop instruments, all while minimizing the use of toxic solvents and extra-column volume in order to uphold the principles of green chemistry. Together, these developments enable high-performance, on-site chromatographic analysis in diverse and challenging field environments.

##### Transportable MS, LC, and GC

Recent advances in analytical instrumentation have enabled rapid, on-site chemical analysis by integrating MS and chromatography with miniaturized and portable sampling techniques (Table 2). MS technologies are classified into beam-based (sector, time-of-flight, quadrupole) and trapping-based (ion trap) analyzers [122]. Among these, Ambient Mass Spectrometry (AMS) has revolutionized direct analysis, allowing the rapid detection of analytes in complex matrices without extensive sample preparation or chromatographic separation [144,145]. Representative ionization techniques include Low-Temperature Plasma (LTP), miniFAPA, Desorption Electrospray Ionization (DESI), and Direct Analysis in Real Time (DART), with DESI relying on electrospray mechanisms and DART on Atmospheric Pressure Chemical Ionization (APCI). A wide array of other ESI- and APCI-related methods, such as paper spray, touch spray, CBS, and Probe ElectroSpray Ionization (PESI), further extend the analytical capabilities of AMS [146,147,148,149,150,151,152,153,154,155,156]. DESI has been already used for airborne particulate monitoring: dynamic DESI sampling of filter-mounted urban aerosols enabled the quantification of nitro-PAHs down to 0.1 ng m^−3^, correlating concentration hotspots with traffic patterns and seasonal biomass burning events [145]. The integration of AMS with microextraction techniques has been achieved through devices such as the Transmission Module™ (TM) and SPME-TM, which use polymer-coated stainless-steel meshes to concurrently isolate and enrich analytes while minimizing interferences that could suppress or enhance ionization. Coupled with high-resolution instruments such as AccuTOF-DART™, this approach produces results comparable to traditional GC-MS within seconds [157,158,159,160]. On-site chemical analysis is further facilitated by miniature MSs, including SPIMS 2000 and systems from BaySpec Inc. (San Jose, CA, USA), enabling the rapid profiling of VOCs and other target compounds in field conditions [160,161]. Parallel developments in chromatography complement these advances. Portable and transportable SPME and Twister applications allow direct coupling to conventional or fast GC systems, improving sample throughput and analytical efficiency. Commercial high-speed GC systems, such as Trace 1300 (Thermo Fisher, Waltham, MA, USA), Nexis GC 2030 (Shimadzu, Kyoto, Japan), Clarus SQ 8 (PerkinElmer, Waltham, MA, USA), and Agilent (Santa Clara, CA, USA) Intuvo 9000, enable ultra-fast analysis and facilitate on-site environmental and industrial hygiene applications. Similarly, compact high-pressure LC systems provide versatile, portable solutions for in-field analysis. These instruments allow precise separation with minimal laboratory footprint and energy consumption, particularly when coupled with MS for real-time detection [162]. Miniaturized systems such as the MiD platform (Microsaic Systems, Sand Hutton, England) integrate chip-based MS detection with LC, enabling efficient direct analysis of complex samples in the field, chemical reaction monitoring, or environmental pollutant assessments. These technologies reduce the need for laborious sample preparation and long chromatographic separations, streamline workflows, and expand the capacity for environmental, industrial, and occupational monitoring under real-world conditions.

##### Man-Portable MS, GC, and LC Systems

Nowadays, modern portable instruments allow rapid, high-resolution measurements of gases, vapors, and liquids directly in the field (Table 3). Membrane Introduction Mass Spectrometry (MIMS) utilizes membrane sampling probes to allow the selective permeation of gaseous or aqueous molecules into the mass spectrometer for qualitative and quantitative analysis. As MIMS does not require prior sample preparation, it is ideally suited for portable MS systems and on-site applications [163,164,165,166]. MIMS does not require energy support to power mechanical pumps (rotary or turbomolecular) to achieve a vacuum; at most it can compensate for the use of these with chemical pumps or ion pumps. Extensions of MIMS, including Trap-and-Release (T&R) MIMS and Single-Sided MIMS (SS-MIMS), enable the sensitive detection of polar Semi-Volatile Organic Compounds (SVOCs) through preconcentration within ultra-thin membranes followed by rapid thermal desorption [167,168,169,170,171,172]. In 2002, Fiber Introduction Mass Spectrometry (FIMS) was introduced, coupling SPME directly to MS. FIMS uses PDMS-coated fibers to achieve selective adsorption, efficient preconcentration, and single-sided desorption of VOCs and SVOCs, enabling high-throughput analysis at trace levels [173]. Commercial portable systems implementing these technologies include VapourSense Q-Technologies’ portable quadrupole MS and 1st Detect’s MMS-1000™, a miniature ion trap MS with a rapid response and an integrated pre-concentrator for enhanced sensitivity. Parallel developments in portable GC have expanded field capabilities. Portable GC and GC-MS systems, including backpack-style instruments such as HAPSITE, Teledyne FLIR Griffin G510, Torion T-9, and EXPEC-3500, allow rapid on-site measurements of gases, vapors, and airborne pollutants, with response times as low as one second. These systems often combine MEMS-based pre-concentrators, SPME fibers, and miniaturized columns to reduce sample preparation and enhance sensitivity; for example, Torion T-9 was applied on field evaluations of roadside air emissions, reporting LODs of 0.02–0.05 µg m^−3^ for BTEX, during continuous 5 min sampling cycles at 30 km h^−1^ vehicle speed [174]. FLIR’s Griffin G510 pairs a high-speed, low-thermal mass GC column with a linear quadrupole MS, operating in two modes, Survey (real-time vapor screening) and Confirmation (split/splitless injection of vapors, liquids, or solids via SPME/Touch-And-Go probes), enabling the comprehensive detection of chemical agents, industrial toxicants, and environmental pollutants. In ambient air trials, the G510 achieved LODs of 0.05–0.10 µg m^−3^ for BTEX in <4 min Survey scans (100 mL min^−1^ flow) and confirmed semi-volatile PAHs at sub-ng m^−3^ levels after 15 min thermal desorption runs [175]. Miniaturized and portable high-pressure LC systems complement these gas-phase analyses. Advances in column miniaturization, including capillary and micro-chip formats, combined with low-flow pumps and sensitive detection, allow portable LC systems such as Focus LC Compact (Axcend^®^) to perform on-site separation and analysis with minimal solvent consumption. While early portable LC relied on conventional columns and detectors, recent developments integrate UV absorption or MS for detection, enabling field-based chemical monitoring and reaction analysis [176,177,178,179,180]. These innovative transportable instruments reduce laborious sample preparation, enable near real-time measurements, and facilitate the monitoring of dynamic chemical processes across diverse matrices, including gases, vapors, liquids, and complex environmental or industrial samples, combining laboratory-grade performance with field portability. Despite the advantages of on-site monitoring, portable instruments have inherent limitations compared with benchtop systems, including shorter columns (which reduce chromatographic resolution) and mass analyzers with reduced performance. METs therefore serve as front-end concentrators necessary to compensate for the lower intrinsic sensitivity of portable instrumentation.

## 3. Conclusions

The METs coupled with portable chromatographic and MS platforms potentially enable high-fidelity, on-field measurements of airborne pollutants within the exposome framework. By integrating sampling, preconcentration, and analysis into compact, automated workflows, MET-GC, MET-LC, and portable MS systems deliver sub-ppb detection limits, rapid turnaround, and minimal solvent use, thereby meeting the dual demands of green analytical chemistry and exposome-scale exposure assessment. Although progress has been made, bringing these tools into everyday sampling environments will depend on their ability to perform reliably under tough conditions, such as extreme temperatures, high humidity, mechanical shocks, and regular vibrations. To move from prototypes to networks that can truly support epidemiological work, it is equally important to focus on affordable manufacturing, the use of open-source hardware and software, and clear, standardized protocols for calibration, data management, and consistency across devices. Looking forward, the convergence of MET-based preconcentration, ambient ionization MS, microfluidic LC modules, and wireless telemetry promises fully instrumented exposome observatories. This review provides an evaluation of the fourteen commercial METs available for in situ environmental sampling of VOCs, highlighting their capabilities to meet short- and long-term sampling requirements in exposomics. Despite significant progress, METs face technical limitations that shape future research directions. There are physical and chemical limits to miniaturization in order to maintain a high analytical performance. In addition, long-term stability and calibration drift management pose a significant challenge, particularly in extreme environmental conditions. Although METs are mature technologies ready for field use, challenges remain regarding protocol standardization and calibration in extreme environmental conditions (humidity and temperature), which can cause drift in results. The transition to a comprehensive monitoring network will require the greater production of independent validation data and the acceptance of microextraction methods by regulatory bodies in order to overcome the current regulatory gap. Future priorities will therefore include developing new energy solutions to support the integration of unmanned systems, as well as enhancing automation and data integration with detection and interpretation systems.

## Figures and Tables

**Table 1:** Overview of commercially available METs with their respective specifications, materials, and manufacturers. List of acronyms used in the table: Sorbent types: Polydimethylsiloxane (PDMS); Polyacrylate (PA); Polyethylene Glycol (PEG); Divinylbenzene (DVB); Carboxen^®^ (CAR); Hydrophilic–Lipophilic Balanced (HLB); Polyacrylonitrile (PAN); Strong Cation Exchange (SCX); Strong Anion Exchange (SAX); Carbon Molecular Sieve (CMS); Porous Polymer Sorbent (PPS); Activated Carbon (AC); Carbon Wide Range (CWR). GC Injection Port: Thermal Desorption Units (TDUs); On-Port Solvent-Assisted Injection (OPSI); Sorbent Pen™ Desorption Unit (SPDU). All cells containing the label Not Available indicate that the corresponding information is not publicly available online. * Exact numbers depend on coating chemistry, sorbent, matrix, extraction/desorption solvents, thermal cycles, and mechanical handling.

	Name Producer	Design Characteristics	Extraction Phase			GC Injection Port	Automation	Number Of Reuse Cycles *
			Area [mm^2^]	Volume [µL]	Sorbent			
Planar METs	Thin-Film SPME (TF-SPME) (Gerstel Mülheim an der Ruhr, Germany)	Thin film/sheet (40 × 4.85 × 0.04 mm)	198	200	PDMS-PDMS/DVB-PDMS/CAR-PDMS/HLB	TDUs	Off-line manual; on-line with autosamplers	>50–100 extraction–desorption cycles (20–50 uses unless cleaning steps)
	Coated Blade Spray (CBS) (Restek Corp., Bellefonte, PA, USA)	Stainless-steel blade with bonded sorbent. 42 mm length	30	Not Available	PAN/HLB	OPSI	On-line with autosamplers	Single-digit to low double-digit reuses in complex matrices. Dozens in clean matrices
In-tube METs	Needle Trap Device (NTD) (Innoteg, München, Germany)	Gauge 22 or 23 length 50–70 mm	Not Available	Not Available	CAR/DVB	TDUs	On-line with autosamplers	Dozens in dirty matrices, up to >1000 extraction/desorption cycles in clean conditions
	SPDE^TM^ (Chromtech, Bad Camberg, Germany)	Gas-tight 2.5 mL. Needle coating 50 µm	Not Available	4.5	PDMS	Split/Splitless	On-line with autosamplers	No universally defined cycle count
	NTME (Pas Tech, Carolina, Puerto Rico)	Gauge 22 or 23 length 50–80 mm	3	Not Available	Tenax^®^-PDMS-DVB-Carbopack-CAR-CMS	Split/Splitless	Off-line manual; on-line with autosamplers	No universally defined cycle count
	Needlex (Shinwa Chem Ind. Ltd., Kyoto, Japan)	Needle-style extractor with packed/coated interior; i.d. 0.5 mm–o.d. 0.7 mm–length 85 mm	3	Not Available	Tenax^®^-PDMS-DVB-Carbopack-CAR	Split/Splitless	Off-line manual; on-line with autosamplers	20–30 cycles with conditioning after each analysis
	Sorbent Pen (Entech Inst., Simi Valley, CA, USA)	Pen-style sorbent trap; direct desorption to GC/TD. 6.1 mm o.d. length 88.9 mm	Not Available	10	Tenax^®^-Tenax^®^/Carbopack-PDMS/Tenax^®^-PDMS/Tenax^®^/Carbopack-CAR-Carbopack-PDMS/Carbopack	SPDU-5800	Off-line manual; on-line with autosamplers	Optimized for quantitative single-sample (diffusive sampling from 8 h to 2 weeks) collection
	ITEX (CTC Analytics AG, Zwingen, Switzerland)	Syringe-based trap extraction; trap is flash-heated for TD to GC. ITEX syringe 133 µL	3	Not Available	Tenax^®^-Carbopack-CMS-CAR	Split/Splitless	On-line with autosamplers	Reusable and cleaned in-line but with no universally defined cycle count
	MEPS (SGE Analytical Science, Melbourne, Australia)	Packed sorbent bed inside syringe/needle path; micro-SPE format. Syringe 100–250 µL removable needle	<1.0	Not Available	C18-C2-Silica-C8-C8/SCX-SAX	Split/Splitless	Off-line manual; on-line with autosamplers	40–100 samples in many applications but have a limited lifetime
	PowerSorb^®^ (Action Europe, Sausheim, France)	Disposable universal adsorber; cylindrical shape (length: 20 mm, diameter: 2 mm); individually packaged under inert atmosphere	125.7	62.8	PPS	TDUs	Compatible with automated TD/GC systems	Single-use adsorbent polymer
	Getxent tubes (Getxent-Biodesiv LLC, Valence, France)	Tubes dimensions: 8 × 35 mm/0.3 × 1.4 in. Weight: 0.93 g	Not Available	Not Available	Polymer-based odor capture and release	Not applicable, primarily used for odor detection training and not for chemical analysis	Not applicable	No data available
Coated Bar–Disk	Twister (SBSE) (GERSTEL, Mülheim an der Ruhr, Germany)	10–20 mm length	Not Available	63–126	PDMS	TDUs	Off-line manual; on-line with autosamplers	From ten to hundreds after conditioning
	Monotrap (MMSE) (GL Sciences, Tokyo, Japan)	Porous silica monolith trap/disk with high surface area. Diameter 2.9–10 mm	45–160	Not Available	AC/C18-C18-GRAPHITE/C18-GRAPHITE/PDMS	Optic-4	Off-line manual; on-line with autosamplers	Single use with chemical extraction; reusable with thermal desorption but subject to thermal degradation
	Magic Chemisorber (Frontiers Laboratories, Fukushima, Japan)	Length 6.0 mm (500 µm), 34.5 mm (500 µm), and 30.0 mm (100 µm)	15	4.5	PDMS/PEG	TDUs	Off-line manual; on-line with autosamplers	Reusable by thermal desorption; no universally defined cycle count
SPME METs	SPME (Thermo/Supelco/Restek)	Fused silica fiber with bonded sorbent coatings. Conventional 100 µm × 10 mm coating–0.7 mm o.d.	9.4–40	0.6–0.9	PDMS, CAR/PDMS, DVB/CAR/PDMS, PEG, etc.	Desorb directly in GC inlet/TDUs	Off-line manual; on-line with autosamplers	From 30 to >200 (matrix-dependent)
	SPME-Arrow Thermo/Agilent/Restek	Arrow housing with a thicker sorptive phase for greater capacity (phase lengths typically 20 mm). 1.1–1.5 needle o.d.	44–62.8	3.8–11.8	PDMS-PA-CWR/PDMS-PDMS/DVB-DVB/CWR-PDMS	Conventional (liner 2.0 mm i.d.)	Off-line manual; on-line with autosamplers	Reusable but with no universally defined cycle count (matrix-dependent)
	Hi-Sorb (Markers Int., Bridgend, Wales (UK))	Standard (8 cm) or short (4 cm) length	65	65	PDMS-PDMS/DVB-PDMS/CWR-DVB/CWR/PDMS	HiSorb extraction module	Off-line manual; on-line with autosamplers	Reusable but with no universally defined cycle count (matrix-dependent)

**Table 2:** Overview of commercially available transportable GC, LC, and MS with their respective specifications and applications.

		Name of Producer	Design Characteristics	Weight	Dimensions	Sample Format/Injection	Control Type	Applications
GC	1	Falcon ULTRAFAST GC Teledyne(Thousand Oaks, CA, USA).	Ultra-fast GC; mountable on a vehicle/mobile bench	11 kg	43 × 28 × 22 cm	Gas, direct injection	Software + display	Biogas, hydrocarbons.
	2	AccuChrome^TM^ GC Galvanic Applied Sciences (Calgary, AB, Canada)	GC for natural gas; robust and transportable— many models available	36.6 kg	32 × 28 × 18 cm	Gas stream sampling and C6–C9 measurements	Analyzer software/PLC integration; continuous process monitoring	Natural gas, industrial process, custody transfer, refinery/process monitoring, emissions compliance.
	3	Mobil GC RotaChrom Technologies (Budapest, Hungary)	Multi-detector GC; transportable on a mobile bench	11 kg	36 × 30 × 20 cm	Direct sampling	Expert System and software	Biodiesel, oil.
	4	Trace 1300 GC Thermo Scientific (Waltham, MA, USA)	Modular GC with Instant-Connect plug-in injector and detector modules	35 kg	44 × 67 × 45 cm	Liquid injections, gas sampling and support special adapters.	Controlled via PC/chromatography software	Environmental, petrochemical.
	5	Nexis™ GC-2030 Shimadzu (Kyoto, Japan)	Modular GC with “Analytical Intelligence” features: self-diagnostics, instrument monitoring, automatic functions	Not published reliably	Not published reliably	Capillary liquid injection, gas sampling	Controlled via LabSolutions software. Touch panel, system support remote monitoring and control	Environmental, petrochemical, chemical, food, VOC.
	6	Clarus SQ 8 PerkinElmer (Shelton, CT, USA)	PerkinElmer’s GC front end (Clarus 580 or Clarus 680) with an SQ 8 MS	Different models: 26–50 kg	50 × 32 × 77 cm	Liquid injections. Support headspace and thermal desorption sample introduction. Vapors, volatile compounds	TurboMass GC/MS software, allowing integration with sample introduction modules (headspace, thermal desorbers)	Environmental, food safety, forensic, pharmaceutical.
	7	Intuvo 9000 Agilent (Santa Clara, CA, USA)	Microfluidic flow path architecture: uses Flow Chips/Guard Chips and microfluidic connectors for leak-free, ferrule-free connections	32 kg	51 × 27 × 69 cm	Liquid injection (split/splitless). Gas sampling via valves (gas sampling loops integrated)	GC instrument firmware interface. Controlled and integrated with chromatography data system software	Environmental, VOC analysis. General chemical, petrochemical.
LC	1	MicroLC SCIEX (Framingham, MA, USA)	Compact UHPLC; integrated autosampler; UV/MS detector	18 kg	50 × 40 × 30 cm	Liquid, autosampler	Software Analyst	Bioanalysis, proteomic.
	2	S 600 TreVenLab (San Fior, Italy)	Modular HPLC; multiple gradients; UV/DAD detector	15 kg	45 × 35 × 30 cm	Liquid, autosampler	Software PC	Environmental, quality check, research.
	3	S 500 TreVenLab (San Fior, Italy)	Modular compact HPLC series (analytical/micro/semi-prep)	Not specified	Not specified	Liquid; autosampler optional (60-position)	Software PC	Environmental, quality check, research.
	4	Waters Arc HPLC Waters (Framingham, MA, USA)	Compact HPLC; compatible with legacy systems; UV/PDA detector	22 kg	60 × 45 × 40 cm	Liquid, autosampler	Empower software	Bioanalysis.
	5	JASCO LC 4000 Series JASCO Europe (Cremella, Italy)	Modular HPLC; UV/VIS, RI, DAD detector	18 kg	50 × 40 × 30 cm	Liquid, autosampler	ChromNAV software	Environmental.
	6	Selekt Biotage (Uppsala, Sweden)	High-performance automated flash LC system	25 kg	54 × 33 × 39 cm	Liquid; flash cartridges/columns (automatic switching between columns), fraction collector	15” touchscreen, Biotage software (touch + PC connectivity)	Preparative/flash purification, synthetic chemistry, rapid method purification.
	7	ECOM Flash LC ECOM (Pully, Switzerland)	Compact preparative/flash LC systems and compact HPLC modules	Not specified	Not specified	Liquid; cartridge/flash columns; fraction collection	PC/DataApex/Clarity integration (software control)	Preparative chemistry, flash purification, organic synthesis labs.
	8	i-Series/i-Series Plus Shimadzu (Kyoto, Japan)	Integrated compact HPLC/UHPLC line	Not specified	Not specified	Liquid; highly accurate autosampler options (0.1–50 µL, etc.)	LabSolutions software; touchscreen, PC options, remote control	Pharmaceutical QC, environmental, routine lab.
	9	ACQUITY Arc Waters (Framingham, MA, USA)	Compact UHPLC	59 kg	62 × 57 × 57 cm	Liquid; autosampler; multi-column managers (up to 15 column switching options)	Empower CDS (Waters)	Regulated labs, pharmaceutical method modernization, bio/pharma, QC.
	10	AccuTOF^TM^ LC DART^TM^ JOEL (Peabody, MA. USA)	High-resolution time-of-flight MS with an atmospheric pressure DART (Direct Analysis in Real Time) ion source	Not specified	Not specified	Gases, vapors or surfaces in front of the DART ion source. Solids, liquids, placing sample directly into the DART ionization region	JOEL’s software	Rapid/direct analysis of drugs. Food, flavor, fragrance. Forensics, homeland security. Environmental screening of VOCs contaminants.
MS	1	PTR 3c PTR-TOF-MS Kore Technology (Ely, Cambridgeshire, UK.)	Transportable PTR-TOF-MS	150 kg	92 × 73 × 53 cm	Direct gas injection (air/VOC stream)	Fully computer-controlled operation, automatic analysis software function	Ambient air VOC monitoring, industrial monitoring, cleanroom contamination control, food and flavor analysis and security.
	2	SPIMS 2000 Hexin Mass Spectrometry (Guangzhou, China)	Second level multi-component monitoring system of atmospheric VOCs	Not specified	Not specified	VOC analysis	Software	Emergency monitoring, daily inspection and pollution portrait.
	3	4500 MID^®^ Microsaic Systems (York, England)	Combines the vacuum system, electronics and computer inside one box.	32 kg	55 × 35 × 25 cm	Liquid, autosampler	Masscape^®^ Clarity, PrepCon, Remote Operations Protocol	Emergency monitoring, daily inspection and pollution portrait.

**Table 3:** Overview of commercially available portable GC, LC and MS with their respective specifications and applications.

		Name of Producer	Design Characteristics	Weight	Dimensions (L × W × H)	Sample Format/Injection	Control Type	Applications
GC	1	Torion T-9 PerkinElmer (Waltham, MA, USA)	GC-MS with ion trap, built-in battery, touchscreen	14.5 kg	38 × 30 × 18 cm	SPME, headspace, syringe	Touchscreen, software	Environmental screening, VOCs, forensics, security.
	2	TRIDION-9 FLIR Systems (Wilsonville, ON, USA)	Compact GC-MS built for rapid screening; battery operation	15 kg	40 × 35 × 20 cm	SPME, headspace	Touchscreen, software	Defense, emergency chemical detection, first responders.
	3	HAPSITE ER INFICON (Bad Ragaz, Switzerland)	Person-portable GC-MS	19 kg	46 × 43 × 18 cm	Air probe, direct injection, canisters; supports SPME accessories	Onboard display (6.5”), PC software	Environmental forensics, security.
	4	Micro GC Fusion INFICON (Bad Ragaz, Switzerland)	Modular micro-GC (2–4 module chassis)	2 modules: 6.2 kg	2 modules: 46 × 19.6 × 25.4 cm	Gas streams—direct gas injection or pre-concentrator options	PC software	Industrial/process gas analysis, environmental on-site gas analysis.
	5	GC-5000 AMA Instruments (Campogalliano, Italy)	Compact on-line/field GC for continuous BTEX/VOC monitoring	12.5 kg	Not available	Loop/loop injection, automated sampling; PID or FID options	Onboard software, PC interface, display	Air quality and industrial site monitoring.
	6	GC 866 Chromatotec (Saint-André de Cubzac, France)	Auto-GC for VOC/BTEX, mobile air cabinets installation.	22 kg	60 × 48.2 × 22.2 cm	Loop injection/thermal desorption options/automatic sampling	Onboard controller, PC software; remote comms (Ethernet/RS-232)	Regulatory ambient BTEX monitoring.
	7	Teledyne Falcon Ultrafast GC Teledyne Analytical Instruments (City of Industry, CA, USA)	Ultrafast GC designed for compact bench/field use; small footprint and light weight	11 kg	43 × 22 × 28 cm	Gas samples; direct injection or headspace/preconcentration depending on configuration	Onboard electronics, PC control	Rapid VOC/hydrocarbon screening, biogas monitoring.
	8	NGC 8206 ABB (Zurich, Switzerland)	Field GC for natural gas, multidetector	10–15 Kg	40 × 35 × 22 cm	Direct gas stream sampling (N2–C6+ analysis)	ABB software/field interface (digital I/O).	Natural gas custody, field analyzer, energy sector.
	9	Portable GC-MS 3500S Expec Technology (Hangzhou, China)	Portable, rugged design suitable for field use	20 kg	44 × 43 × 22 cm	Software-switchable single mass spectrometry injection; GC-MS internal standard sorbent tube injection; GC-MS internal standard quantitative ring injection; headspace injection; needle injection; SPME	PC software, touchscreen	Environmental monitoring, food safety, biomedicine, forensics and toxicology.
	10	TGI-1100 Shanghai Huaai (Shanghai, China)	Portable GC (FID/TCD options)	10 kg	33 × 27 × 19 cm	Direct injection, FID/TCD options	PC software, LCD/display; field controllers available	Industrial gas monitoring, emergency response, outdoor VOCs.
LC	1	CroMini LC-80 TreVenLab (San Fior, Italy)	All-in-one compact HPLC	Not specified	Not specified	Liquid samples, manual injection (loop); autosampler optional	Windows software (PC control)	Teaching, clinical chemistry, routine QC.
	2	Advion Interchim Compact Advion Ltd.(Ithaca, NY, USA)	Portable LC-MS	18 kg	50 × 40 × 30 cm	Liquid (LC/infusion)—needs LC front-end for standard HPLC workflows	Touchscreen, PC software; simplified interfaces for benchtop use	Pharmaceutical R&D, metabolomics, environmental, QC (bench-portable LC-MS).
	3	Focus LC Micro LC^®^ AXCEND (Bengaluru, Karnataka, India)	Compact and portable HPLC	8 kg	30 × 20 × 20 cm	Manual and automated injection with Auto Focus	Efficient control, acquisition, and reporting with Axcend Drive software	Pharmaceuticals, environmental testing and education, chemical manufacturing.
MS	1	QGA 2.0 Hiden Analytical (Warrington, England)	Benchtop MS for real-time analysis of gases	30 kg	58 × 34 × 42 cm	Accepts gases and vapors; can be used with various inlets optimized for different sampling methods	Supplied with QGAsoft software for quantitative analysis, and MASsoft Professional for instrument control, data handling, and external I/O	Environmental monitoring, gas production and storage.
	2	HPR-20 OEMS—Hiden Analytical (Warrington, England)	On-line MS for electrochemical cells/battery testing (heated capillary, etc.).	14 kg	40 × 30 × 22 cm	Gas/vapor sampling	PC software and remote control	Fuel cells, batteries, on-line gas analysis.
	3	FLIR Griffin G510 FLIR Systems (Wilsonville, ON, USA)	Rugged GC-MS instrument; options for vehicle mounting.	19 kg	50 × 40 × 30 cm	Syringe, SPME, headspace, vapor probe	Touchscreen, software; field libraries	Defense, security, environmental rapid screening.
	4	Advion Interchim Compact MS Advion Ltd. (Ithaca, NY, USA)	Compact ESI-MS; simplified interface; transportable	32 kg	28 × 66 × 56 cm	Liquid, direct injection and multiple sample introduction modes (TLC plate reader, ASAP)	Touchscreen, software	Pharmaceutical, environmental sector, metabolomics.
	5	Portable Mass Spectrometry BaySpec (San Jose, CA, USA)	Portable MS solution. Portability series	10 kg	36 × 33 × 22 cm	Pulsed atmospheric sampling	Touchscreen interface	Ideal for fieldwork and applications requiring a high degree of mobility and versatility.

## Data Availability

No new data were created or analyzed in this study. Data sharing is not applicable to this article.

## Abbreviations

The following abbreviations are used in this manuscript:

METs	Microextraction Technologies
HAPs	Hazardous Air Pollutants
VOCs	Volatile Organic Compounds
GCs	Gas Chromatographs
LCs	Liquid Chromatographs
MSs	Mass Spectrometers
PM	Particulate Matter
FAMs	Field Analytical Methods
AQS	Air Quality System
IUPAC	International Union of Pure and Applied Chemistry
SPME	Solid-Phase Microextraction
TWA	Time-Weighted Average
HLB	Hydrophilic–Lipophilic Balance
CBS	Coated Blade Spray
NTME	Needle Trap Microextraction
LOD	Limit of Detection
MMSE	MonoTrapTM—Monolithic Material Sorptive Extraction
AMS	Ambient Mass Spectrometry
LTP	Low-Temperature Plasma
DESI	Desorption Electrospray Ionization
DART	Direct Analysis in Real Time
APCI	Atmospheric Pressure Chemical Ionization
PESI	Probe Electrospray Ionization
TM	Transmission Module
MIMS	Membrane Introduction Mass Spectrometry
SVOCs	Semi-Volatile Organic Compounds
FIMS	Fiber Introduction Mass Spectrometry
TF-MET	Thin-Film Microextraction Technologies
TF-SPME	Thin-Film Solid-Phase Microextraction
PDMS	Polydimethylsiloxane
PA	Polyacrylate
PEG	Polyethylene Glycol
DVB	Divinylbenzene
CAR	Carboxen^®^
HLB	Hydrophilic–Lipophilic Balanced
PAN	Polyacrylonitrile
SCX	Strong Cation Exchange
SAX	Strong Anion Exchange
CMS	Carbon Molecular Sieve
PPS	Porous Polymer Sorbent
AC	Activated Carbon
CWR	Carbon Wide Range
TDUs	Thermal Desorption Units
OPSI	On-Port Solvent-Assisted Injection
SPDU	Sorbent Pen™ Desorption Unit
